# In Vivo Parieto-Occipital White Matter Metabolism Is Correlated with Visuospatial Deficits in Adult DM1 Patients

**DOI:** 10.3390/diagnostics12102305

**Published:** 2022-09-24

**Authors:** Stefania Evangelisti, Laura Ludovica Gramegna, Silvia De Pasqua, Magali Jane Rochat, Luca Morandi, Micaela Mitolo, Claudio Bianchini, Gianfranco Vornetti, Claudia Testa, Patrizia Avoni, Rocco Liguori, Raffaele Lodi, Caterina Tonon

**Affiliations:** 1 Department of Biomedical and Neuromotor Sciences, University of Bologna, 40138 Bologna, Italy; 2 Functional and Molecular Neuroimaging Unit, IRCCS Istituto delle Scienze Neurologiche di Bologna, 40139 Bologna, Italy; 3 Department of Experimental, Diagnostic and Specialty Medicine, University of Bologna, 40138 Bologna, Italy; 4 Department of Physics and Astronomy, University of Bologna, 40127 Bologna, Italy; 5 UOC Clinica Neurologica, IRCCS Istituto delle Scienze Neurologiche di Bologna, 40139 Bologna, Italy

**Keywords:** MR spectroscopy, metabolism, myotonic dystrophy, white matter, visuospatial functions

## Abstract

Myotonic dystrophy type 1 (DM1) is a genetic disorder caused by a (CTG) expansion in the DM protein kinase (*DMPK*) gene, representing the most common adult muscular dystrophy, characterized by a multisystem involvement with predominantly skeletal muscle and brain affection. Neuroimaging studies showed widespread white matter changes and brain atrophy in DM1, but only a few studies investigated the role of white matter metabolism in the pathophysiology of central nervous system impairment. We aim to reveal the relationship between the metabolic profile of parieto-occipital white matter (POWM) as evaluated with proton MR spectroscopy technique, with the visuoperceptual and visuoconstructional dysfunctions in DM1 patients. MR spectroscopy (3 Tesla) and neuropsychological evaluations were performed in 34 DM1 patients (19 F, age: 46.4 ± 12.1 years, disease duration: 18.7 ± 11.6 years). The content of neuro-axonal marker N-acetyl-aspartate, both relative to Creatine (NAA/Cr) and to myo-Inositol (NAA/mI) resulted significantly lower in DM1 patients compared to HC (*p*-values < 0.0001). NAA/Cr and NAA/mI correlated with the copy of the Rey-Osterrieth complex figure (r = 0.366, *p* = 0.033; r = 0.401, *p* = 0.019, respectively) and with Street’s completion tests scores (r = 0.409, *p* = 0.016; r = 0.341, *p* = 0.048 respectively). The proportion of white matter hyperintensities within the MR spectroscopy voxel did not correlate with the metabolite content. In this study, POWM metabolic alterations in DM1 patients were not associated with the white matter morphological changes and correlated with specific neuropsychological deficits.

## 1. Introduction

Myotonic dystrophy type 1 (DM1) is a multisystem disease associated with an unstable expansion of nucleotide repeats (CTG [cytosine–thymine–guanine] triplets) in the genes coding for myotonic dystrophy protein kinase (*DMPK*) [1] that contribute to the accumulation of mutant RNA aggregates, with consequent mis-splicing of downstream effector genes that affect almost all cells and organs of the human body [2,3,4]. Therefore, the disease is characterized by multisystem involvement with predominantly muscle and brain affection.

Brain involvement in DM1 includes the presence of hyperostosis and whole brain atrophy, mostly evaluated in group analysis using advanced brain MRI techniques [5], and subcortical atrophy in form of increased ventricular volume that rarely can result in a severe cerebral ventriculomegaly with a normal pressure hydrocephalus-like appearance on MR imaging [6].

White matter involvement in DM1 has now been irrefutably demonstrated by a large body of literature that evidenced the presence of T2/fluid-attenuated inversion recovery (FLAIR) hyperintensities preferentially located bilaterally in frontal, temporal (temporo-polar in one-third of the cases) [5], and parietal lobes at periventricular and subcortical locations [7]. Advances in imaging technology recently provided substantial evidence for a diffuse involvement of the normal appearing white matter (NAWM) in DM1 patients, as demonstrated mainly by diffusion tensor imaging (DTI) techniques with mean diffusivity (MD) values increase and fractional anisotropy (FA) values reduction [5].

Brain proton MR spectroscopy (^1^H-MRS) is a non-invasive technique that can provide an in vivo evaluation of the biochemical profile within specific volumes of interest (VOI). It has been shown that a reduction of N-acetyl-aspartate (NAA) level is related to neuronal and/or axonal damage or loss as in neurodegenerative disorders, and an increase in myo-Inositol (mI) is considered a marker of glial reaction. Other metabolites that are typically quantified are creatine (Cr) and choline-containing compounds (Cho), respectively, markers of cellular energy and of cellular proliferation, increased membrane turnover, or inflammation [8]. MRS allows us to detect brain metabolic abnormalities that can be present at early stages of the diseases, when the structural morphological alterations are not evident, and findings of morphological MR imaging are still absent or ambiguous.

Few previous studies, summarized in Supplementary Table S1, described brain metabolic profile in DM1 patients through the ^1^H-MRS technique [9,10,11,12,13,14], with heterogeneous technical parameters, VOI selection in regions including both gray and white matter and patients’ populations. The most common finding from previous studies is a reduction of NAA both as relative and absolute concentrations.

Several studies have demonstrated that DM1 patients show a selective impairment in cognitive functioning, particularly concerning attention, memory, executive, and visuospatial domains [2,15,16,17,18,19]. Visuospatial functioning refers to cognitive processes necessary to “identify, integrate, and analyze space and visual form, details, structure and spatial relations in more than one dimension” [20]. Visuospatial processing, therefore, results from the integration of three main processes: 1. perception of basic visual elements such as light, contrasts, and orientation, principally mediated by the occipital cortices; 2. visual construction, resulting from the progressive integration of visual percepts with input from the parietal, temporal, and frontal cortices, and 3. visual memory (recall/recognition of visual information and topographical memory) [21]. Visuospatial deficit also impacts visuoconstructional abilities, which require the coordination of fine motor skills with spatial abilities to copy well-planned and organized geometric figures [22].

Both visuospatial and visuo-constructional impairments are considered distinctive features of the DM1 cognitive profile [23,24,25] and have been proposed as prognostic factors of cognitive and structural brain progressive degeneration [19,26]. Moreover, subjects with DM1 frequently present psychiatric comorbidities embracing personality and/or mood disorders, and a characteristic emotional imbalance [27,28]. In spite of their cognitive/affective difficulties and progressive motor impairment, patients with DM1 also may present anosognosia (or “lack of insight”), a mental disorder that impairs the ability to understand and perceive accurately one’s own illness [15,17,28]. The lack of disease awareness may seriously hinder early diagnostic assessment as it leads to secondary misattribution of symptoms and low compliance to treatment [17]. To date, the inability to be aware of one’s own medical condition has been correlated in DM1 patients to white matter alterations detected by morphological MR imaging [17,29].

The aim of the current study was to depict the metabolism of parieto-occipital white matter through the ^1^H-MRS technique in DM1 patients, while investigating the role of white matter pathology in neuropsychological dysfunction.

## 2. Materials and Methods

### 2.1. Participants

We enrolled thirty-seven consecutive adult patients with a genetic diagnosis of myotonic dystrophy type 1 (DM1 or “Steinert’s disease”) (F/M 22/15, age: 46.8 ± 11.7 years). Patients were recruited at the Neuromuscular Centre of the UOC Neurological Clinic of the IRCCS Institute of Neurological Sciences, Bologna, IT, during the period from 1 Junaury 2019 to 31 December 2021. The study protocol was approved by the local Ethical Committee (n. 1088-2020-OSS-AUSLBO).

CTG triplet expansion sizes were measured in all patients in genomic DNA extracted by peripheral blood leukocyte using Southern blot analysis. Depending on the number of repeat expansions patients were classified into three genetic classes: E1 (50-150 CTG repeats), E2 (150-1000 CTG repeats), and E3 (more than 1000 CTG repeats). 

Depending on the age of disease onset, patients were stratified into three groups: congenital/childhood onset (from birth to 10 years of age), juvenile/adult onset (from 11 to 40 years of age), and late onset (more than 40 years of age). All DM1 patients underwent standardized clinical, neuropsychological, and MR spectroscopy and imaging evaluations.

A cohort of ten sex- and age-matched healthy control subjects (HC) was also recruited for the quantitative MR evaluation. They were selected from the database of the Neuroimaging Laboratory, designed to collect normative values of MR parameters for clinical and research purposes. Demographic features of patients and controls are reported in Table 1.

### 2.2. Neuropsychological Evaluation

An extensive neuropsychological assessment was performed on all patients. However, in the present study, we focus specifically on general cognitive functioning in addition to visuospatial and visuoconstructional abilities. Thus, each participant underwent a neuropsychological examination to obtain a clinical profile that included a general cognitive screening test (Mini-Mental State Examination, MMSE [30]) and a test assessing culture-free logical reasoning (Raven’s Colored Progressive Matrices Test, CPM-47 [31]).

Visuospatial abilities were investigated by The Benton Judgment of Line Orientation Test (BJLOT [32]) and the Street’s Completion Test [33]. The copy of the Rey-Osterrieth complex figure (ROCF-copy [34]) further explored visuoconstructional abilities. Patients’ raw scores were corrected according to Italian normative values [21,35]. Percentages of impairment of DM1 patients who showed significant neuropsychological dysfunctions across different cognitive domains were established using Italian normative data for both, score adjustment (sex, age, and education) and the definition of cut-off thresholds.

Finally, levels of self-awareness (or the presence of anosognosia) were measured with the Measurement of Anosognosia Instrument [36]. This questionnaire consists of a series of dichotomous items assessing the patients’ performance in selected cognitive domains (i.e., attention, memory, language, executive functions) and in daily life settings and was administered with both self-report and informant-rating versions. The responses provided by the patient in the double modality were compared to quantify the number of discrepant answers. Discrepancy scores were used to quantify the presence of anosognosia [37,38].

### 2.3. Brain MR Protocol

Participants underwent a multimodal standardized brain MR protocol acquired with a 3T scanner (Siemens MAGNETOM Skyra) equipped with a high resolution 64-channel coil.

The protocol included single-voxel proton MR spectroscopy (suppressed-waterPoint RESolved Spectroscopy, PRESS) technique within the parieto-occipital white matter (POWM, volume = 8 mL, echo time/repetition time TE/TR = 30/2000 ms, number of averaged fids = 64, duration ~2 min) [39], a volumetric T1-weighted sequence (3D MPRAGE, magnetization prepared rapid gradient-echo, sagittal acquisition, isotropic voxel 1 × 1 × 1 mm^3^, no slice gap, TE = 2.98 ms, TR = 2.300 ms, Inversion Time IT = 900 ms, flip angle = 9°, acquisition matrix = 256 × 256, pixel bandwidth = 240 Hz, GRAPPA acceleration factor = 2, duration ~5 min) and a volumetric fluid-attenuated inversion recovery (FLAIR) T2-weighted sequence (3D SPACE, sagittal acquisition, isotropic voxel 1 × 1 × 1 mm^3^, no slice gap, TE = 428 ms, TR = 5000 ms, IT = 1.800 ms, flip angle = 120°, acquisition matrix = 256 × 256, pixel bandwidth = 780 Hz, GRAPPA acceleration factor = 2, duration ~5 min). 

### 2.4. Neuroradiological MRI Evaluation

All MRI images were analyzed by a neuroradiologist with 8 years of experience in reporting neurodegenerative disorders who evaluated the presence of structural brain MRI alterations in DM1 patients, including the presence of white matter changes appearing as hyperintensities on T2- weighted sequence and normal pressure hydrocephalus-like appearance.

In particular, the severity of white matter changes appearing as hyperintensities on the T2- weighted sequence was estimated using a modification of the age-related white matter change rating scale (Fazekas scale) [40]. This scale is scored from 0 to 3 as follows: (0) *Absent*, absence of periventricular or subcortical lesions (a single punctate hyperintensity less than 5 mm in size within the subcortical and periventricular white matter was considered normal); (1) *Slight*, continuous periventricular lines less than 5 mm in length and/or various non-confluent subcortical white matter foci less than 5 mm in size; (2) *Moderate*: continuous periventricular lines 5 to 10 mm long and/or subcortical white matter foci 5 to 10 mm in size beginning to merge; and (3) *severe*: periventricular stripes more than 10 mm long and/or irregular confluent lesions more than 10 mm in size. 

Regarding the presence of the normal pressure hydrocephalus-like appearance, the z- EVANS index was calculated based on the ratio between the maximum z-axial length of the frontal horns and the maximum cranial z-axial length on the coronal plane, which was perpendicular to the anteroposterior commissure plane on the anterior commissure as previously described [41]. Similarly, the callosal angle, considered the angle of the roof of the lateral ventricles on the coronal plane at the posterior commissure level [42] was reported. The presence of hyperostosis, enlarged perivascular spaces, and incidental MRI findings were reported as well.

### 2.5. Whole Brain White Matter Changes Analysis

Patients’ white matter changes appearing as white matter hyperintensities on FLAIR T2-weighted images were quantified through Jim software (Version 7.0, Xinapse Systems, Northants, UK, http://www.xinapse.com (accessed on 22 July 2022)), a semi-automatic threshold-based method specific for white matter lesions load segmentation. 

FLAIR T2-weighted images were linearly (FLIRT, part of the Oxford FMRIB Software Library FSL) co-registered to the T1-weighted images and the transformation matrix was then used to align the lesion map to the T1-w image. In this way, the accuracy of brain tissue segmentation could be improved by reducing the intensity contrast in T1-w images within known WM lesions by using the FSL lesion filling tool [43,44]. T1-weighted images were also non-linearly (FNIRT) aligned to the MNI (Montreal Neurological Institute) template, and the transformation was applied to the lesion maps and a group map was built by summing individual maps together. 

### 2.6. Proton MRS

#### 2.6.1. Spectra Quality Assessment and Analysis Method

Assessment of spectra quality was based on visual inspection, evaluating the absence of artifacts and the baseline, along with the signal to noise ratio (SNR ≥ 14) and Full Width at Half Maximum (FWHM ≤ 3.7) evaluation. Examples of the measured spectra can be seen in Figure 2D. 

Spectra were analyzed with the fitting software LCModel, version 6.3 [45]. N-Acetyl-aspartate (NAA), choline (Cho)-containing compounds, and myo-Inositol (mI) content was evaluated relative to creatine (Cr) or mI as internal references. As a measure of post processing model fitting quality, LCModel estimated fitting error <15% was considered.

#### 2.6.2. White and Gray Matter Segmentation in the MRS VOI

Estimates of the tissue fractions within the MRS voxels was performed for each subject by using FSL-MRS (Figure 2B) (in particular, Siemens DICOM spectral data were converted into NIfTI with *spec2nii tool*, then *svs_segment* was applied after that the lesion-filled T1-weighted image was segmented into grey matter (GM), white matter (WM) and cerebrospinal fluid (CSF), with the tool *fast* [46]. In this way, potential bias related to the different tissue-volume fractions can be excluded, being more methodologically accurate and of support for the interpretation [47]. Moreover, the amount of WM lesion volume that was included within the MRS voxel was also assessed (Figure 2C), in order to calculate the amount of normal-appearing vs. altered WM included in the metabolic profile evaluation.

### 2.7. Statistical Analysis

The normality of data distributions was assessed using the Shapiro-Wilk test. When normal continuous variables were compared between two groups, *t*-tests were used (or ANCOVA when covariates of no interests and/or more than two groups were included in the model), otherwise, Wilcoxon tests were applied. In MRS data comparisons, age and sex were added as covariates of no interest.

Categorical variables were compared with chi-square tests. Pearson’s or Spearman’s correlations were performed according to the distribution. The statistical analyses were conducted with R-software version 3.5.2 (https://www.r-project.org/ (accessed on 22 July 2022)). Data from patients belonging to the E3 genetic class are reported for information, but not included in group comparison analysis given that only two patients belonged to this class. In order to evaluate the accuracy of proton MR spectroscopy parameters in discriminating DM1 patients from HC, a receiver operating characteristics (ROC) curve analysis was performed. The optimal cutoff value corresponded to the higher Youden’s index.

## 3. Results

### 3.1. DM1 Sample Characteristics

Among the 37 recruited DM1 patients, one did not complete the MR protocol and two had suboptimal quality spectra (due to motion artifacts, related lipids contamination from the scalp, and imprecise VOI localization). Therefore, 34 good quality spectra were included in the analysis, along with all 10 spectra from HC. Among the patients included, four had congenital/childhood onset, 22 juvenile/adult onset, and eight late had onset. According to the CTG repeats, 13 of them belonged to E1, 19 to E2, and two to E3 classes. Main descriptive data for patients and HC are reported in Table 1.

### 3.2. Neuropsychological Results

Considering the whole cohort of DM1, the general cognitive profile assessed by MMSE was impaired in 11.8% (4/34) of them (mean corrected score ± sd 26.5 ± 2.7, range 18.7–30.0), mostly driven by E2 (15.8%; 26.6 ± 2.8 18.7–30.0) and E3 (50%; 21.9 ± 4.2; 18.9–24.9) pathological scores. 

The deficit in logical reasoning, namely the ability to reason organized spatial perceptions into systemically related whole (CPM-47) was found in 14.7% (26.4 ± 5.7; 10.8–36.0) of DM1 patients, affecting E2 (15.8%; 25.9 ± 5.3; 14.8–33.3) and all E3 patients (14.1 ± 4.7; 10.8–17.4), while E1 mean performance ranked within a normal range (28.8 ± 3.6; 23.7–36.0).

Both visuospatial and visuoconstructional functions were remarkably impaired in all DM1 patients, with a performance following a worsening trend across the three classes of nucleotide triplet’s expansion. Specifically, inaccurate judgments of lines orientation (BJLOT) were assessed in 26.5% (9/34) (22.5 ± 7.2; 6.0–30.0) of the patients, that is 15.4% of E1 (24.9 ± 6.1; 10.0–30.0), 26.3% of E2 (22.5 ± 6.3; 8.0–30.0), and 100% E3 (7.5 ± 2.1;6.0–9.0). Moreover, 16/34 that is 47.1% (27.3 ± 7.8; 8.0–36.0) of them ((23.1% of E1 (29.8 ± 7.4; 8.0–36.0), 57.9 % of E2 (27.3 ± 6.2; 8.0–34.6), and 100% of E3 (10.8 ± 2.5; 9.1–12.5) failed to accurately copy a complex geometrical figure (ROCF-copy), which is suggestive of deficits in motor planning and visuoconstructional abilities. Finally, deficits in visual perceptual organization, as measured with SCT, were found in only 5/34 that is 5.9% of DM1 patients (6.4 ± 2.3; 1.3–10.2), pertaining to E1 (7.7%; 5.4 ± 2.2; 1.3–8.6) and E2 (5.3%; 7.4 ± 1.9; 3.1–10.3) classes.

Finally, discrepancy scores between self- and informant-ratings of patient’s cognitive performance were observed in most of the DM1 cohort (20/34, 58.8%), where 17 out of 34 patients (50%) reported an overestimation of their deficit, while 3 out of 34 (8.8%; −1.3 ± 2.1; −5.0–3.0) reported a lack of awareness (anosognosia). The over- vs. underestimation ratio differed among DM1 classes: 7.7% of E1 (−1.0 ± 2.0; −4.0–3.0) patients presented anosognosia, while 53.9% of them overestimated their own deficits; the same trend was accentuated among E2 patients (10.5%, −1.3 ± 2.3; −5.0–2.0); underestimating patients versus 31.6% overestimating), while both E3 patients showed to overestimate their cognitive impairments.

Neuropsychological findings are summarized in Table 2. When neuropsychological data were analyzed comparing patients pertaining to different age of onset groups, the main differences were observed between patients with a congenital onset and those with a juvenile one, essentially concerning worse general cognitive abilities (MMSE), logical reasoning (CPM-47) and visuospatial abilities (BJLOT) among the congenital onset DM1 patients (see Supplementary Table S2 for more details).

### 3.3. Neuroradiological Results

The extensive neuroradiological characterization based on conventional morphological MR images for the DM1 cohort is reported in Table 3.

Overall, almost all DM1 patients (30/34, 88.2%) had a Fazekas-scale score greater than zero, and 18/34 (52.9%) presented white matter hyperintensities on the FLAIR T2- weighted sequence in the temporo-polar regions. Hyperostosis was present in 11/34 (32.4%) patients, mainly within the frontal regions, and only in four was diffuse. Cerebral ventriculomegaly was observed in the majority of patients (19/34, 55.9%) and was symmetrical in all except two. The z- EVANS index was on average 0.28 ± 0.04 and 6/34 (17.6%) patients also presented disproportionately enlarged subarachnoid-space hydrocephalus (DESH) [48]. On average the callosal angle was (112.8° ± 16.6°), and all the patients had an enlarged perivascular space within the hemispheric white matter. 

**Figure 1:**
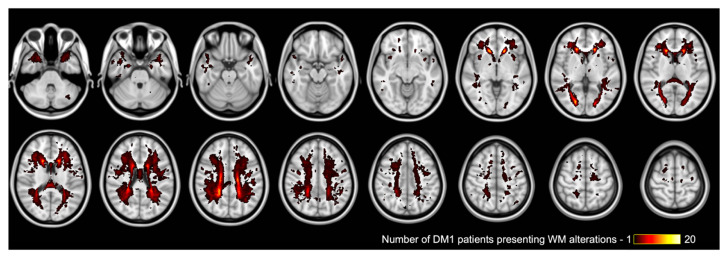
Group-level WM lesion maps in DM1 patients, overlayed on the standard MNI (Montreal Neurological Institute) template. The values of the displayed maps (range 1–20) correspond to the number of patients that presented WM alterations at ach specific locations on FLAIR T2-weighted images.

As for the whole brain white matter lesion load evaluation, it was performed in 33 over 34 patients since one (n.33) did not have good-quality FLAIR T2-weighted images due to movement artifacts. In the whole group of DM1 patients, the total lesion load was 7883 ± 12,347 mm^3^ (mean ± SD), 3732 [8474] mm^3^, (median [IQR]), 89–67,863 mm^3^ (range). When the three genetic classes were instead considered separately, it was (4301 ± 4574) mm^3^ (2995 [7085], 208–14,283 mm^3^) in E1 patients, (10184 ± 15,551) mm^3^ (4835 [10,232], 89–67,863 mm^3^) in E2 patients and 10,617 mm^3^ in the E3 patient. Even if a larger white matter lesion load seems to be present in E2 patients if compared to E1, this difference was not statistically significant (Wilcoxon test, *p*-value = 0.2231). To represent the overall spatial distribution of white matter changes in our DM1 cohort, the group lesion map is shown in Figure 1.

### 3.4. Proton MRS

**Figure 2:**
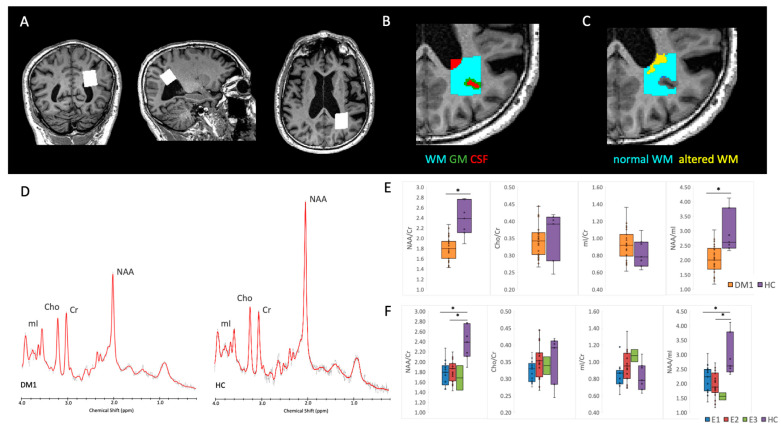
Top: representative ^1^H-MRS 8 mL volume of interest localization within the parieto-occipital white matter of a DM1 patient (**A**), with the corresponding segmentation of WM, GM and CSF within the MRS VOI (**B**) and distinction between normal-appearing and altered WM within the MRS VOI (**C**). Bottom: ^1^H-MRS study results, with the comparison of a spectrum from a DM1 patient and an age and sex-matched HC (**D**), box-plot of metabolites content for DM1 versus HC group (**E**) and for each DM1genetic class E1, E2, E3 versus HC group (**F**). NAA: N-Acetyl-aspartate, Cr: creatine, Cho: choline, mI: myo-inositol. (*) statistically significant at *p* < 0.05.

NAA/Cr and NAA/mI resulted in significantly lower in DM1 patients compared to HC (Table 4, Figure 2D,E). As for the genetic subgroups, no significant differences were found between E1 and E2 patients, but the ANCOVA highlighted a significant effect of group for NAA/Cr and NAA/mI due to the significant alterations in both E1 and E2 patients compared to HC (Table 4, Figure 2F). An analogous effect was observed when DM1 patients were stratified according to the age of onset: no significant differences were found between the subgroups of patients, but only compared to HC (Supplementary Table S3). 

The fraction of different tissues (WM, GM, and CSF) included within the MRS volume of interest (Figure 2B) did not differ significantly between groups (Table 5, Supplementary Table S4), excluding therefore a potential bias in the comparisons of metabolites content.

As for the WM fraction included in the MRS VOI (Figure 2C), the ratios of normal-appearing WM and altered WM on FLAIR T2 weighted- images were instead different between the two classes E1 vs. E2 (Table 5). We therefore verified whether it was correlated with the metabolites content, and there were no correlations of the percentage of altered WM within the MRS VOI and NAA/Cr (Spearman rho = −0.064, *p* = 0.725), Cho/Cr (rho = 0.251, *p* = 0.158), mI/Cr (rho = 0.133, *p* = 0.461), NAA/mI (rho = −0.151, *p* = 0.401). These results suggest that the metabolic alterations evaluated with MRS are not related to the structural alteration of the tissue, but instead present also in normal-appearing WM.

### 3.5. Correlations

NAA/Cr within POWM showed significant positive correlations with ROCF-copy (r = 0.366, *p* = 0.033) and performance Street’s completion test (r = 0.409, *p* = 0.016) scores. NAA/mI was also positively correlated with ROCF-copy (r = 0.401, *p* = 0.019) and with Street (r = 0.341, *p* = 0.048) tests scores. Moreover, both NAA/Cr and NAA/mI negatively correlated with disease duration (r = −0.530, and *p* = 0.001; r = −0.468, *p* = 0.005, respectively). The total WM lesion load was negatively correlated with the ROCF-copy test score (r = −0.360, *p* = 0.040,), whereas no significant correlations between metabolite content and total WM lesion load were found.

Given the significant differences, NAA/Cr and NAA/mI were the selected parameter to evaluate ^1^H-MRS diagnostic accuracy. For NAA/Cr, the ROC curve analysis showed an area under the curve (AUC) of 0.94, with sensitivity and specificity respectively of 90% and 94% when considering a cut-off value of 2.101. As for NAA/mI, the AUC was 0.88, with sensitivity and specificity respectively of 100% and 65% when considering a cut-off value of 2.281. At the single-subject level, considering NAA/Cr, only two patients had unaltered values (Table 3).

## 4. Discussion

Our study demonstrates that analysis of brain metabolism in the parieto-occipital white matter by proton magnetic resonance spectroscopy (^1^H-MRS) may assist in the neuroradiological diagnosis of DM1 by revealing pathological NAA/Cr ratio with high sensitivity and specificity. Moreover, this finding expanded previous studies performed by using other advanced MRI techniques such as tract-based spatial statistics (TBSS), and region of interest diffusion tensor imaging (ROI-based DTI) suggesting that alterations in white matter metabolism may be associated with deficits in visuospatial and visuoconstructional abilities, confirming involvement of the normal-appearing parieto-occipital white matter.

Altered NAA/Cr as measured with ^1^H-MRS within the parieto-occipital white matter could identify DM1 patients with good accuracy, sensitivity, and specificity, although it was not significantly different between the genetic classes E1 and E2.

Interestingly, for three patients this metabolic alteration was the only sign detected in the context of an apparently normal morphological brain MRI examination. This approach is based on a non-invasive and reproducible technique, with a relatively short acquisition time, that could be included in the clinical neuroradiological routine [8].

White matter alterations in DM1 are typically hyperintense changes in the FLAIR T2-weighted images. The pathophysiology of the WM involvement, as for the pathological alterations that underly the imaging phenotypes, are still under debate [2].

A recent review evaluated 41 pathological studies focusing on the microscopic brain alteration in a total of 130 DM1 patients [49]. Eight studies highlighted the presence of gliosis in the white matter in 37 DM1 patients overall, although the type of gliosis was mostly not specified (e.g., reactive astrogliosis, microgliosis/activated microglia cells, or oligodendrocyte response). The largest study among these eight included 11 patients and reported that a loss of axons accompanied the myelin loss [50], whereas in two case reports, myelin loss coincided with “relative axonal sparing” [51,52]. Moreover, Itoh et al., also found capillary hyalinization and fibrillary gliosis [50]. Interestingly, heterotopic neurons were found in white matter in three studies [53,54,55] that evaluated nine patients. Four brains pertained to patients with congenital DM1 that died shortly after birth; the other five were from DM1 patients. Heterotopic neurons were found both in brain subcortical and deep white matter. There are a few hypotheses on the pathogenetic mechanism of the observed histopathological alterations and, therefore, of the MRI signal alterations. Some studies have found the presence of abnormal mutant RNA accumulation in the nuclei of brain cells including oligodendrocytes [56,57]. It has been suggested that these foci of RNA accumulation are toxic for the cells as they compromise the regulation of alternative splicing in different cells, thus providing a possible link between the molecular pathophysiology of DM1 and visible histopathological alterations [56]. Additionally, Renard et al. proposed increased burden due to microvascular changes and lack of drainage of interstitial fluid and degraded protein products as a mechanism for anterior temporal white matter lesions in DM1 [58].

A key aspect of the present study is that the ^1^H-MRS VOI was placed for most of the patients within normal-appearing white matter (NAWM). The metabolic alterations detected by MRS are therefore present also in the NAWM, suggesting that they are either not related to the structural alteration of the tissue or appear before the morphological tissue damage. In fact, the proportion of normal-appearing and altered white matter within the investigated tissue by ^1^H-MRS were evaluated and did not correlate with the metabolites content suggesting that the white matter metabolic alterations in DM1 patients were not associated with morphological alterations of the same volume of interest.

Widespread involvement of the NAWM in DM1 patients had already been shown by diffusion MR studies, that found altered diffusivity parameters also within brain regions not affected by the hyperintensities [5]. Our results support this evidence, also from a metabolic perspective.

Overall, our results are in line with the reduction of NAA levels that was described in previous MRS studies in gray matter and white matter of DM1 patients. In particular, lower NAA level was shown in patients with congenital and juvenile myotonic dystrophy, supporting the hypothesis of a developmental disorder of neurons in the brain [9,13]. Lower NAA levels have been also shown at an early stage of the disease [11], and in both DM1 and DM2 patients [12]. 

More heterogeneous results are instead reported for the other metabolite content. Depletion of Cr and Cho levels, particularly in the frontal white matter, were found in DM1 patients and these changes in brain metabolites can differentiate DM1 and DM2, despite the similar structural MRI alterations, suggesting differences in their underlying pathophysiological mechanism [12]. Significant abnormalities in brain metabolites, especially higher mI and Cr, were also previously found, with a positive correlation with CTG repeat size [10]. Finally, evidence of brain (pathological accumulation of lactate) and skeletal muscle impairment of oxidative metabolism in DM1 patients has been also described through the MRS technique [14].

Previous proton MRS studies mainly focused on localization within the gray matter, such as the parietal cortex [9], midoccipital and temporo-parietal gray matter [10,12], insular cortex [11], anterior cingulate gyrus, and basal ganglia [13]. Only two studies also placed VOIs within the WM, focusing on the frontal WM [12,13]. The involvement of parieto-occipital regions in DM1 patients was previously demonstrated with morphological MRI [5,43] but no correlations with the neuropsychological data were reported. With the present results, we propose the parieto-occipital white matter as a crucial region of interest also for ^1^H-MRS, without necessarily including any WM lesions but focusing on the NAWM.

Moreover, NAA alterations within POWM correlated with neuropsychological tests evaluating visuospatial and visuoconstructional functions, all features considered as recurrent in DM1 neuropsychological profile [16,17]. Specifically, positive correlations between NAA levels and neuropsychological functions showed that a decrease in NAA metabolites in parieto-occipital WM is bound to a worsening of the visuospatial and visuoconstructional performance. Moreover, a large part of our DM1 cohort presented a misperception by excess or by default (anosognosia) of the disease’s impact on their everyday life’s cognitive skills. Differently from the results presented by Baldanzi et al. [17], in our study, only a subgroup (8,82%) of our cohort presented a proper anosognosia. Those differences might be due to the different scales for anosognosia used in both studies, each one measuring different aspects of awareness. On the one hand, lack of disease awareness appears to be typically related to global brain atrophy in neurodegenerative disorders [59], and fMRI resting state studies suggested that self-awareness is supported by two closely interconnected neural networks, the “default mode network” (DMN) and the “attention system”, encompassing both frontal lobes and parietal structures [60,61]. On the other hand, visuospatial functioning is heavily reliant on the integrity of the parietal lobe and its connections with the occipital regions [62,63,64]. Neuroimaging studies in DM1 patients showed consistently a substantial impairment in visuospatial and visuoconstructional abilities that were significantly correlated with WM abnormalities and cortical volume loss as demonstrated by advanced morphological and diffusivity techniques such as voxel-based morphometry VBM and TBSS [5,18,26,65,66,67,68]. Here we showed that these abilities are also related to metabolic alterations that can be present within the NAWM. We focused on the WM involvement, from the metabolic and morphologic point of view, but, although DM1 patients have a widespread distribution of atrophy, a contribution from specific grey matter regions cannot be excluded, consistently with previous neuroimaging studies [5,18,67,68].

In conclusion, white matter metabolic changes highlighted by MR spectroscopy technique in adult DM1 patients while not being expression of tissue structural damage can be related to specific neuropsychological deficits.

## Figures and Tables

**Table 1:** Main characteristics of DM1 patients (as a whole cohort, and according to the genetic class and the age of onset and HC.

	**DM1**			**HC**	***p*-Value**
**N**	34			10	-
**Sex F/M**	19/15			2/8	0.07
**Age, years**	46.4 ± 12.1 (22.3–71.0)			41.5 ± 19.4 (19.8–75.4)	0.48
**Disease duration, years**	18.7 ± 11.6 (0.6–47.9)			-	-
	**E1**	**E2**	**E3**		***p*-Value**
	**(50–150 CTG Repeats)**	**(150–1000 CTG Repeats)**	**(More than 1000 CTG Repeats)**		
**N**	13	19	2		-
**Sex F/M**	6/7	12/7	1/1		0.14
**Age, years**	50.2 ± 10.4 (28.1–71.0)	43.8 ± 13.0 (22.9–67.8)	46.3 ± 13.3 (36.8–55.7)		0.49
**Disease duration, years**	14.2 ± 9.5 (0.6–29.3)	17.9 ± 9.2 (6.4–35.5)	43.5 ± 9.2 (36.8–47.9)		0.29
	**Congenital/Childhood**	**Juvenile/Adult**	**Late**		***p*-Value**
	**(from Birth to 10 Years of Age)**	**(from 11 to 40 Years of Age)**	**(More than 40 Years of Age)**		
**N**	4	22	8		-
**Sex F/M**	2/2	11/11	6/2		0.59
**Age, years**	37.5 ± 13.0 (25.3–55.7)	43.2 ± 10.0 (22.9–57.4)	59.5 ± 6.8 (52.9–71.0)		0.0004 *
**Disease duration, years**	34.0 ± 12.2 (21.3–49.7)	19.2 ± 8.3 (7.2–35.5)	6.5 ± 4.6 (0.6–13.1)		<0.0001 *

Age and disease duration are reported as mean ± standard deviation (range). (*) statistically significant at *p* < 0.05.

**Table 2:** Neuropsychological data in DM1 patients (as a whole cohort and according to the genetic class).

**Cognitive Domain**	**Tests**	**All DM1**						
		**Corrected Score Mean ± sd (Range)**		**Cut Offs**		**Pathological Scores (%)**		
**Cognitive screening**	MMSE	26.5 ± 2.7 (18.7–30.0)		≤23.8		11.8		
**Non-verbal Intelligence**	CPM-47	26.4 ± 5.7 (10.8–36.0)		≤18.9		14.7		
**Visuoperception**	BJLOT	22.5 ± 7.2 (6.0–30.0)		≤18		26.5		
	SCT	6.4 ± 2.3 (1.3–10.2)		≤2.3		5.9		
**Visuoconstructional abilities**	ROCF-copy	27.3 ± 7.8 (8.0–36.0)		≤28.9		47.1		
**Anosognosia**	Measurement of Anosognosia Instrument	−1.3 ± 2.1 (−5.0–3.0)		-		8.8		
**Cognitive Domain**	**Tests**	**E1**		**E2**		**E3**		***p*-Value**
		**Corrected Score Mean ± sd (Range)**	**Pathological Scores (%)**	**Corrected Score Mean ± sd (Range)**	**Pathological Scores (%)**	**Corrected Score Mean ± sd (Range)**	**Pathological Scores (%)**	
**Cognitive screening**	MMSE	27.1 ± 1.6 (24.8–30.0)	0	26.6 ± 2.8 (18.7–30.0)	15.8	21.9 ± 4.2 (18.9–24.9)	50.0	0.19
**Non-verbal Intelligence**	CPM-47	28.8 ± 3.6 (23.7–36.0)	0	25.9 ± 5.3 (14.8–33.3)	15.8	14.1 ± 4.7 (10.8–17.4)	100	0.14
**Visuoperception**	BJLOT	24.9 ± 6.1 (10.0–30.0)	15.4	22.5 ± 6.3 (8.0–30.0)	23.3	7.5 ± 2.1 (6.0–9.0)	100	0.33
	SCT	5.4 ± 2.2 (1.3–8.6)	7.7	7.4 ± 1.9 (3.1–10.3)	5.3	3.6 ± 0.8 (3.0–4.1)	0	0.017 *
**Visuoconstructional abilities**	ROCF-copy	29.8 ± 7.4 (8.0–36.0)	23.1	27.3 ± 6.2 (8.0–34.6)	57.9	10.8 ± 2.5 (9.1–12.5)	100	0.29
**Anosognosia**	Measurement of Anosognosia Instrument	−1.0 ± 2.0 (−4.0–3.0)	7.7	−1.3 ± 2.3 (-5.0–2.0)	10.5	−2.0	0	0.73

(*) statistically significant at *p* < 0.05. MMSE: Mini-Mental State Examination, CPM-47: Raven’s Colored Progressive Matrices, BJLOT: Benton Judgment of line orientation test-h version, SCT: Street’s completion test, ROCF: Rey-Osterrieth complex figure.

**Table 3:** Neuroradiological characteristics for DM1 patients.

**Patient** **Number**	**Sex**	**Age**	**Onset**	**Genetic** **Class**	**Fazekas Scale Score**	**Temporo-Polar Fazekas Scale Score**	**Hyperostosis**	**Hyperostosis** **Distribution**	**Normal Pressure Hydrocephalus-like Appearance**							**Incidental Brain Findings**	**^1^** **H-MRS**	
									**Cerebral** **Ventriculomegaly**	**Maximum Axial Length** **of the Frontal Horns (mm)**	**Maximum Cranial** **Axial Length (mm)**	**z-Score EVANS**	**DESH**	**Callosal Angle (°)**	**Enlarged Perivascular** **Spaces**		**NAA/Cr**	**Altered**
1	M	28	Juvenile/adult	E1	1	0	0		0	22.2	95.0	0.23	0	107	1	0	1.961	1
2	F	39	Juvenile/adult	E1	1	0	0		1	24.0	80.6	0.29	0	99	1	Periventricular gray matter heterotopy	1.804	1
3	M	40	Juvenile/adult	E1	1	0	0		1	28.5	92.7	0.30	0	111	1	Retrovermian arachnoid cyst	1.793	1
4	M	46	Juvenile/adult	E1	1	1	0		0	20.8	83.3	0.25	0	122	1	0	1.608	1
5	F	50	Juvenile/adult	E1	1	0	1	Frontal	0	20.0	80.7	0.25	0	118	1	0	1.855	1
6	M	52	Juvenile/adult	E1	2	1	0		1	35.5	82.2	0.43	1	63.5	1	0	1.751	1
7	M	52	Juvenile/adult	E1	1	1	1	Diffuse	1	30.0	92.3	0.32	0	102	1	0	1.455	1
8	F	53	Juvenile/adult	E1	2	1	0		0	21.5	84.3	0.25	0	121	1	0	1.465	1
9	F	53	Late	E1	1	1	0		1	24.1	92.7	0.26	0	126	1	0	2.033	1
10	F	55	Late	E1	0	0	0		0	21.0	90.0	0.23	0	130	1	Left parietal microbleed	1.899	1
11	F	56	Late	E1	1	0	0		1	26.4	82.1	0.32	0	124	1	0	1.739	1
12	M	57	Juvenile/adult	E1	1	1	0		1	24.8	89.0	0.28	0	116	1	0	1.479	1
13	M	71	Late	E1	2	0	0		1	30.3	84.9	0.36	1	81	1	Left lacunar chronic infarct	2.272	0
14	F	23	Juvenile/adult	E2	0	0	0		1 (asymmetrical)	19.5	83.8	0.23	0	109	1	0	2.095	1
15	M	25	Congenital/childhood	E2	1	0	1	Diffuse	1	27.1	89.1	0.30	0	129	1	0	1.868	1
16	F	30	Juvenile/adult	E2	1	1	1	Frontal	0	25.4	86.1	0.29	0	106	1	0	2.195	0
17	F	32	Congenital/childhood	E2	1	0	1	Diffuse	1	21.9	84.0	0.26	0	130	1	Ectopic pituitary adenoma	1.624	1
18	F	34	Juvenile/adult	E2	1	1	0		0	20.2	84.3	0.24	0	120	1	0	1.914	1
19	F	34	Juvenile/adult	E2	1	1	0		0	20.6	88.4	0.23	0	118	1	0	1.912	1
20	M	35	Juvenile/adult	E2	0	0	0		1	24.6	80.5	0.30	0	110	1	0	1.873	1
21	M	36	Juvenile/adult	E2	1	0	1	Frontal	0	20.0	81.6	0.24	0	122	1	Tumor-like lesion	1.532	1
22	F	43	Juvenile/adult	E2	1	1	1	Frontal	0	20.3	81.6	0.25	0	100	1	0	1.931	1
23	M	43	Juvenile/adult	E2	2	1	0		1 (asymmetrical)	23.8	89.8	0.26	0	112	1	0	1.740	1
24	M	45	Juvenile/adult	E2	1	0	0		0	21.3	79.0	0.27	0	122	1	0	1.967	1
25	F	47	Juvenile/adult	E2	2	1	0		0	20.1	77.8	0.26	0	119	1	0	1.719	1
26	F	51	Juvenile/adult	E2	1	1	1	Frontal	1	24.2	80.5	0.30	0	135	1	0	1.429	1
27	M	53	Late	E2	1	0	0		1	27.5	88.6	0.31	1	130	1	0	1.796	1
28	F	55	Juvenile/adult	E2	0	0	1	Frontal	0	23.3	80.0	0.29	0	116	1	0	1.606	1
29	M	57	Juvenile/adult	E2	3	1	0		1	25.0	85.0	0.29	0	97	1	0	1.760	1
30	F	59	Late	E2	1	1	0		0	21.5	83.2	0.26	0	125	1	0	1.975	1
31	F	61	Late	E2	1	0	0		0	23.2	86.8	0.27	0	139	1	0	2.059	1
32	F	68	Late		2	1	1	Frontal	1	30.2	80.0	0.37	1	80	1	0	1.559	1
33	F	37	Congenital/childhood	E3	1	1	1	Diffuse	1	25.7	83.5	0.30	1	93	1	0	1.928	1
34	M	56	Congenital/childhood	E3	3	1	0		1	28.7	85.3	0.34	1	103	1	Cerebellar malacia, talamic hyperintensity	1.442	1

M: male, F: female, DESH: disproportionately enlarged subarachnoid-space hydrocephalus, ^1^H-MRS: proton magnetic resonance spectroscopy, NAA: N-acetyl-aspartate, Cr: creatine, 0 = absent, 1 = present.

**Table 4:** ^1^H-MRS results in DM1 patients (whole cohort and E1, E2, E3 classes separately) and HC.

	**DM1**			**HC**	***p*-Value**		
**NAA/Cr**	1.80 ± 0.22 (1.43–2.27)			2.32 ± 0.29 (1.89–2.78)	<0.0001 *		
**Cho/Cr**	0.34 ± 0.05 (0.27–0.44)			0.34 ± 0.07 (0.25–0.42)	0.86		
**mI/Cr**	0.92 ± 0.17 (0.61–1.36)			0.83 ± 0.14 (0.63–1.09)	0.15		
**NAA/mI**	2.02 ± 0.46 (1.18–3.04)			2.86 ± 0.61 (2.33–4.12)	<0.0001 *		
	**E1**	**E2**	**E3**		***p*-Value**	**Post-Hoc**	
**NAA/Cr**	1.78 ± 0.24 (1.46–2.27)	1.82 ± 0.21 (1.24–2.20)	1.68 ± 0.34 (1.44–1.93)		< 0.0001 *	E1 vs. HC E2 vs. HC E1 vs. E2	<0.0001 * <0.0001 * 0.88
**Cho/Cr**	0.32 ± 0.03 (0.28–0.38)	0.35 ± 0.05 (0.27–0.44)	0.34 ± 0.04 (0.31-0.37)		0.3663		
**mI/Cr**	0.84 ± 0.14 (0.61–1.18)	0.95 ± 0.18 (0.69–1.36)	1.07 ± 0.10 (1.00-1.15)		0.0737		
**NAA/mI**	2.17 ± 0.48 (1.37–3.04)	1.97 ± 0.44 (1.18–2.72)	1.56 ± 0.17 (1.44–1.69)		0.0003 *	E1 vs. HC E2 vs. HC E1 vs. E2	0.0044 * 0.0001 * 0.51

Data are reported as mean ± standard deviation (range). Data for E3 are reported, but not included in group comparison analysis. (*) statistically significant at *p* < 0.05. NAA: N-Acetyl-aspartate, Cr: creatine, Cho: choline, mI: myo-inositol.

**Table 5:** Tissue fractions within parieto-occipital white matter ^1^H-MRS volume of interest in DM1 patients (whole cohort and E1, E2, E3 classes separately) and HC.

**VOI**	**DM1**			**HC**	***p*-Value**
**% WM**	70 ± 9 (55–89)			77 ± 10 (62–86)	0.07
**% altered WM (§)**	5.4 ± 9.1 (0–45.6) 1.7 [7.5]			NA	-
**% GM**	20 ± 6 (4–32)			15 ± 9 (4–28)	0.11
**% CSF**	9 ± 6 (2–28)			8 ± 4 (2–15)	0.50
**VOI**	**E1**	**E2**	**E3**		***p*-Value**
**% WM**	68 ± 9 (55–83)	72 ± 8 (56–89)	66 ± 12 (58–75)		0.23
**% altered WM (§)**	2.6 ± 5.5 (0–19.2) 0 [1.0]	7.4 ± 10.7 (0–45.6) 3.2 [9.6]	3.2		0.027 * (#)
**% GM**	21 ± 5 (15–30)	20 ± 7 (4–32)	18 ± 1 (17–19)		0.67
**% CSF**	11 ± 8 (2–28)	8 ± 4 (3–21)	16 ± 11 (8–24)		0.23

Data are reported as mean ± standard deviation (range). Data for E3 are reported, but not included in group comparison analysis. (§) Given the not-normal distribution of the variable, also median [interquartile range] are reported. (#) Wilcoxon test *p*-value. (*) statistically significant at *p* < 0.05. WM: white matter, GM: grey matter, CSF: cerebrospinal fluid.

## Data Availability

The data presented in this study are deposited in Zenodo repository (https://zenodo.org (accessed on 12 September 2022)) and are accessible with the following link: https://doi.org/10.5281/zenodo.7068988 (accessed on 12 September 2022).

## Supplementary Materials

The following supporting information can be downloaded at: https://www.mdpi.com/article/10.3390/diagnostics12102305/s1, Table S1: Summary of previous brain ^1^H-MRS studies in DM1 patients; Table S2: Neuropsychological data in DM1 patients according to the age of onset; Table S3: ^1^H-MRS results in DM1 patients according to the age of onset; Table S4: Tissue fractions within parieto-occipital white matter ^1^H-MRS volume of interest in DM1 patients according to the age of onset.
