# Supplementary material for: In Vivo Parieto-Occipital White Matter Metabolism Is Correlated with Visuospatial Deficits in Adult DM1 Patients
Source: Diagnostics (Basel). 2022 Sep 24;12(10):2305. doi: 10.3390/diagnostics12102305 (PMC9600392; doi:10.3390/diagnostics12102305)
Supplement: Supplementary file 1 [file diagnostics-12-02305-s001.zip › diagnostics-1871407-supplementary.pdf]

Supplementary Table S1. Summary of previous brain ^{1}H-MRS studies in DM1 patients.

	Hashimoto et al., 1995 [9]	Chang et al., 1998 [10]	Akiguchi et al., 1999 [11]	Vielhaber et al., 2006 [12]	Takado et al., 2015 [13]	Gramegna et al., 2018 [14]
**Scanner magnetic field**	1.5T	1.5T	1.5T	1.5T	3T	1.5T
**^{1}H-MRS Sequence**	SVS STEAM or SE<br>TE/TR= 270/1500ms	SVS PRESS<br>TE/TR= 30/3000ms	SVS STEAM<br>TE/TR= 19/2500ms	SVS PRESS<br>TE/TR= 135/1500ms	SVS PRESS<br>TE/TR= 30/1500ms<br>MRSI PRESS<br>TE/TR= 144/1500ms	SVS PRESS<br>TE/TR= 288/1550ms
**MRS Localization**	GM (volume 8-27 ml)<br>Parietal (5/5 pts)<br>Left occipital (3/5 pts)<br>Frontal cortex (1 pt)	GM (volume 3-5 ml)<br>Midoccipital<br>Left temporoparietal	GM (volume 27 ml)<br>Insular cortex, included the frontal, temporal, and parietal opercula	GM, WM (volume 4.8-6 ml)<br>Midoccipital and temporoparietal<br>Frontal	GM, WM (volume SVS 4.1ml, MRSI 18×18cm2 or 20×20cm2)<br>SVS: frontal GM (anterior cingulate gyrus)<br>frontal WM<br>MRSI: two 10-mm axial slices through the basal ganglia or the upper lateral ventricles	CSF (volume 4.7-10.1 ml)<br>Lateral ventricles
**Measured metabolites**	NAA, Cho, Cr	NAA, Cho, Cr, mI, Glx	NAA, Cho, Cr	NAA, Cho, Cr	NAA, Cho, Cr, mI, Glu, Gln, Glx	Lac
**Spectra analysis**	Peak height evaluation	Manufacturer platform and semi-automatic software (absolute quantification)	In-house software	LCModel (absolute quantification)	Manufacturer platform and LCModel	JMRUI
**DM1 Cohort size**	5	13	21	14 DM1<br>15 DM2	14	25
**DM1 Diagnosis**	Congenital MD	Clinical diagnosis	Clinical diagnosis (no congenital)	Genetically confirmed	Genetically confirmed	Genetically confirmed (1 congenital DM1)

**CGT repeat length**<br>**mean ± sd (range)**	NA	847 ± 314 (173 - 1434)	CTG expansion confirmed in 3 patients	Range 250–750	685 ± 462	E1 (50-150 CTG repeats): 4 pts<br>E2 (150-1000 CTG repeats): 14 pts<br>E3 (> 1000 CTG repeats): 7 pts
**Age, years**<br>**mean ± sd**	7.3 ± 5.5<br>(range 13 months - 14 years)	37.8 ± 2.7	37.0 ± 13.6	38.8 ± 9.1 (DM1)<br>38.6 ± 7.8 (DM2)	43.6 ± 12.6	39 ± 11
**Disease duration, years**<br>**mean ± sd**	Congenital	13.8 ± 3.5	11.2 ± 7.6	8.5 ± 4.1 (DM1)<br>9.5 ± 6.8 (DM2)	11.8 ± 9.0	16 ± 11
**Age-matched healthy controls**	46 children, 1 adult	24	16	13	13	14
**Investigated correlations**	Age	CTG expansion	Age, MMSE or HDS	Age, age at onset, disease duration, CTG expansion, MMSE	Disease duration, CTG expansion, MMSE, FAB	Age at onset, CTG expansion, DM1 functional scale
**Findings**	• Lower NAA/Cr in all patients except one (13-month- old) also in regions normal at morphological MRI	• Higher mI, Cr and Cho concentration<br>• Positive correlation between mI and Cr levels and CTG repeat size<br>• Trend for NAA level decrease with disease duration	• Lower NAA/Cr and NAA/Cho	• Comparing to healthy controls lower NAA content both in GM and WM in both disease groups<br>• Comparing to healthy controls reduced Cr and Cho in TPGM and FWM only in DM1<br>• No correlations	• MRSI: reduced NAA/Cr in multiple brain regions<br>• SVS: reduced NAA in frontal cortex and frontal WM<br>• Elevated glutamine in the frontal GM<br>• Reduced glutamate in the frontal WM<br>• NAA/Cr in the frontal lobe correlate with CTG repeat length	• 8 patients with pathological accumulation of brain lactate<br>• Compared to those without, larger lateral ventricles, smaller GM volume and higher WM lesion load.
**Interpretation**	Findings suggest a neurodevelopmental disorder.	Findings suggest an increased glial content, more prominent in the midoccipital than in the temporoparietal cortex, related to the severity of disease.	Metabolic changes detected in the younger patients and their slow decline with age suggest a neurodevelopment disorder. No differences in MRS changes between patients with (16/21) and without (5/21) NPS deficits.	Althought structural MRI abnormalities are similar between DM1 and DM2, ^{1}H-MRS is able to differentiate between the two disease subgroups with an overall accuracy of 88%.	Glutamatergic system abnormalities associated with synaptic dysfunction.	Evidence of brain oxidative metabolism deficit.

^{1}H-MRS: proton magnetic resonance spectroscopy, SE=Spin Echo , CTG: cytosine–thymine–guanine, SVS: single voxel spectroscopy, MRSI: magnetic resonance spectroscopy imaging, STEAM: STimulated Echo Acquisition Mode, PRESS: Point RESolved Spectroscopy, NAA: n-acetyl-aspartate, Cho: choline, Cr: creatine, mI: myo-inositol, Glx: Glutamate-Glutamine,Glu: glutamate, Gln: glutamine, , MMSE: Mini-mental state examination, FAB: frontal assessment battery, HDS: Hasegawa Dementia Scale testing.

Supplementary Table S2. Neuropsychological data in DM1 patients according to the age of onset.

Cognitive domain	Tests	Congenital/childhood		Juvenile/adult		Late		P-value	Post-hoc	
		Corrected Score mean±sd (range)	Pathologic al scores (%)	Corrected Score mean±sd (range)	Pathologic al scores (%)	Corrected Score mean±sd (range)	Pathological scores (%)			
**Cognitive screening**	MMSE	23.4±3.4 (19.0-27.0)	50.0	26.9±1.5 (24.8-30)	4.5	27.0±3.8 (19.0-30.0)	12.5	0.010*	Congenital vs juvenile<br>Congenital vs late<br>Juvenile vs late	0.045*<br>0.11<br>0.97
**Non-verbal Intelligence**	CPM-47	16.5±4.3 (10.8-21.3)	75.0	28.2±3.3 (22.9-36)	0	26.0±6.6 (15.0-33.3)	25.0	0.0003*	Congenital vs juvenile<br>Congenital vs late<br>Juvenile vs late	0.0005*<br>0.037*<br>0.17
**Visuopercepti on**	BJLOT	13±6.8 (6.0-21.0)	75.0	24.6±6.2 (8.0-30.0)	13.6	21.6±6.3 (13.0-30.0)	37.5	0.0008*	Congenital vs juvenile<br>Congenital vs late<br>Juvenile vs late	0.0023*<br>0.11<br>0.19
	SCT	4.6±1.3 (3.0-5.9)	0	6.7±2.4 (1.3-10.3)	9.1	6.2±1.7 (3.1-8.0)	0	0.15		
**Visuoconstruc tional abilities**	ROCF-copy	20±10.8 (9.0-30.3)	75.0	28.5± 5.8 (13.8-36)	45.5	27.8±9.7 (8.0-36.0)	37.5	0.06		
**Anosognosia**	Measurement of Anosognosia Instrument	-3.0±1.2 (-4.0 - 2.0)	0	-1.2±2.0 (-5.0-2.0)	4.5	-0.4±2.5 (-4.0 - 3.0)	25	0.32		

(*) statistically significant at p<0.05. MMSE: Mini-Mental State Examination, CPM-47: Raven's Colored Progressive Matrices, BJLOT: Benton Judgment of line orientation test-h version, SCT: Street's completion test, ROCF: Rey-Osterrieth complex figure.

Supplementary Table S3. ^{1}H-MRS results in DM1 patients according to the age of onset.

	Congenital/childhood	Juvenile/adult	Late	P-value	Post-hoc	
**NAA/Cr**	1.72 ± 0.22 (1.44-1.93)	1.77 ± 0.21 (1.43-2.20)	1.92 ± 0.22 (1.56-2.27)	< 0.0001 *	Congenital vs HC	0.0005*
					Juvenile vs HC	< 0.0001*
					Late vs HC	0.0007*
					Congenital vs juvenile	0.97
					Congenital vs late	0.37
					Juvenile vs late	0.28
**Cho/Cr**	0.35 ± 0.02 (0.31-0.37)	0.34 ± 0.04 (0.27-0.44)	0.35 ± 0.06 (0.29-0.44)	0.8864		
**mI/Cr**	0.97 ± 0.20 (0.69-1.15)	0.91 ± 0.18 (0.61-1.36)	0.91 ± 0.13 (0.72-1.10)	0.5348		
**NAA/mI**	1.82 ± 0.39 (1.44-2.36)	2.01 ± 0.48 (1.18-3.04)	2.16 ± 0.44 (1.41-2.65)	0.0002	Congenital vs HC	0.0038*
					Juvenile vs HC	0.0002*
					Late vs HC	0.018*
					Congenital vs juvenile	0.88
					Congenital vs late	0.66
					Juvenile vs late	0.88

(*) statistically significant at $p<0.05$. NAA: N-Acetyl-aspartate, Cr: creatine, Cho: choline, mI: myo-inositol.

Supplementary Table S4. Tissue fractions within parieto-occipital white matter ^{1}H-MRS volume of interest in DM1 patients according to the age of onset.

VOI	Congenital/childhood	Juvenile/adult	Late	P-value
**% WM**	69 ± 8 (58-75)	71 ± 9 (56-89)	69 ± 9 (55-84)	0.31
**% altered WM (§)**	4.7 ± 2.6 (3.2-7.7)<br>3.2 [2.2]	5.5 ± 10.6 (0-45.6)<br>0.4 [6.7]	5.4 ± 6.3 (0-15.8)<br>5.4 [9.0]	0.51
**% GM**	20 ± 4 (17-26)	20 ± 7 (4-32)	21 ± 6 (13-30)	0.46
**% CSF**	11 ± 9 (6-24)	9 ± 6 (2-28)	10 ± 6 (3-21)	0.67

WM: white matter, GM: grey matter, CSF: cerebrospinal fluid.
